# pubassistant.ch: consolidating publication profiles of researchers

**DOI:** 10.12688/f1000research.73493.2

**Published:** 2021-12-20

**Authors:** Reto Gerber, Mark D. Robinson

**Affiliations:** 1 Department of Molecular Life Sciences, University of Zurich, Zurich, 8057, Switzerland; 2 SIB Swiss Institute of Bioinformatics, Zurich, 8057, Switzerland

**Keywords:** open access, publication profiles, R shiny

## Abstract

Online accounts to keep track of scientific publications, such as Open Researcher and Contributor ID (ORCID) or Google Scholar, can be time consuming to maintain and synchronize. Furthermore, the open access status of publications is often not easily accessible, hindering potential opening of closed publications. To lessen the burden of managing personal profiles, we developed a R shiny app that allows publication lists from multiple platforms to be retrieved and consolidated, as well as interactive exploration and comparison of publication profiles. A live version can be found at pubassistant.ch.

## Introduction

Given the increasing number of both researchers and publications as well as publishing modes, ^1^^,^^2^ it becomes a challenge to identify and consolidate all publications from a single author. A few of the main issues are transliteration of names into roman alphabetic system, the non-uniqueness of names, differently written names (e.g., with or without middle initial) and changing affiliation over time. There are broadly speaking two approaches to solve this ambiguity: “unattended” and “attended”. The “unattended” approach tries to automatically resolve ambiguity using additional existing metadata. The “attended” approach relies on human intervention in the form of unique identifiers that enable robust linkage of publications to authors, assuming researchers and their collaborators use them consistently. The most important, widely used and *de facto* standard identifier in many fields is the Open Researcher and Contributor ID (ORCID). ^3^ Other identifiers such as Google Scholar ID ^4^ or ResearcherID (Publons) ^5^ are also used, although they are not as broadly used as ORCID and persistence of identifiers is not always guaranteed. Having multiple identifiers on multiple platforms is not unusual and automatic publication detection and syncing between accounts is possible to some degree. However, automatic synchronization of accounts for different identifiers can be hindered by the fact that not all systems use the standardized DOI (Digital Object Identifier) as document identifier to match publications.

Although the two main standardized identifiers for authors (ORCID) and documents (DOI) are widely adopted, other identifiers are still used, making it often necessary to synchronize publication records on different platforms manually to obtain complete records. For instance, there is no simple one-click solution to synchronize publications between ORCID and Google Scholar. In Google Scholar, publications need to be searched and added manually (if they are not detected automatically) while in ORCID it is possible to input a citation file. A typical workflow to update ORCID based on Google Scholar would therefore be to first search (one by one) in Google Scholar all publications that are listed in ORCID and then add the missing ones. But since it is possible that publications listed in Google Scholar are not in ORCID, the reverse needs to be done to be sure the accounts are up to date. If more accounts need to be synced (e.g., Publons), the complexity and time needed increases accordingly. Although it is possible, and probably advisable, to link accounts for automatic updates (e.g., linking Publons with ORCID), this cannot be done under all circumstances and missing publications are still possible.

While some (commercial) services (such as Dimensions ^6^ or Web of Science ^7^) provide extensive data mining to retrieve publication data, they often also rely on unique identifiers (such as ORCID in the case of Dimensions) for correct assignment. Furthermore, on many platforms that combine different sources, it is not easy to determine where the data originated (e.g., is a publication listed in ORCID or in Publons? or both?). In addition, data exploration and visualization is often restricted to citations over time (except costly commercial services). With the growing awareness, interest and mandates towards Open Science, open access (OA) status of articles can also be of interest. The same is true for preprints, which are not always taken into account despite becoming increasingly important in many research fields. ^8^^,^^9^

Another inconvenience can be the existence of duplicated publications, which can stem either from the association of preprint and peer-reviewed publication or from revisions or different versions. In many cases, it is sensible to treat those closely linked publication as just one publication instead of multiple. If the required information to link publications is missing, automatic detection is not always possible and manual intervention is needed.

Many tools, both commercial and free, exist that allow to explore certain aspects of bibliographies. Furthermore, many of the available tools are not made for individual authors but rather operate on the department, institutions or even country level. Some of the existing commercial tools include Elements (from the company Symplectic ^10^) and Dimensions, both of which are mainly intended for institutional use; but, especially Dimensions also offers functionalities for authors to explore their bibliographies.

Commercial as well as institutional tools provide valuable improvements to the quality of bibliographies, especially in non-STEM subjects where accurate representations of scholars is often more difficult.

Some of the free and/or open tools include Vivo ^11^ (Institutional level, creates ontologies for representing scholarship), Profiles Research Networking ^12^ (Institutional level, help to discover collaborators), ReCiter ^13^ (Institutional level, find publications of authors in PubMed), ImpactStory ^14^ (author level, impact and open access status of publications from ORCID).

Furthermore, tools mainly intended for monitoring open science include the open science monitoring of the European commission ^15^ (country-level), the German open access monitor (institution-level) and OpenAIRE (Open Access Infrastructure for Research in Europe), which provides dashboards (country- or institution-level).

In our case, we took inspiration from the Swiss National Science Foundation’s Open Access Check, ^16^ which allows Swiss researchers to reflect on their publishing practices and encourages various forms of OA, including green OA; importantly, such resources rely on the source databases being up to date in the first place.

To facilitate overview and synchronization of publication records, we provide a web-based application that allows publications for an author to be retrieved from different sources, combines entries, checks for duplicates and downloads citations to easily update records across platforms. Furthermore, the open access status of each publication is provided, which can help to select publications that could be “greened” (i.e., depositing documents in institutional repositories). Taken together, this allows researchers to organize their public publication profiles and to interactively explore the accuracy of records across the various entry points.

## Methods

**Figure 1.**
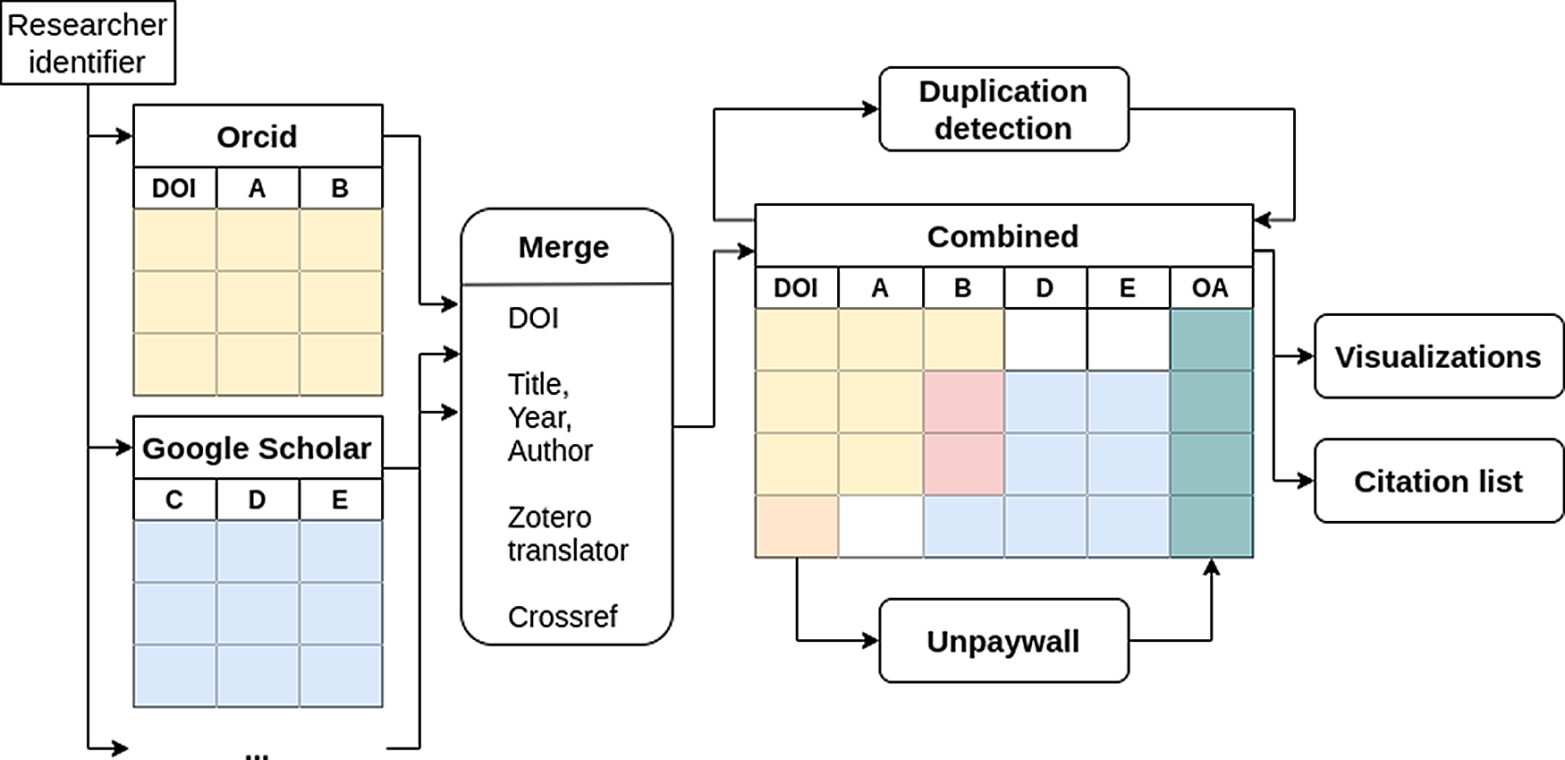
Overview of the data processing. The identifiers given by the user are used to obtain the data from each platform independently. The data is then merged and the open access status (column OA) is obtained using the Digital Object Identifier (DOI). Furthermore duplicates are detected by comparing the titles of the publications.

The workflow is as follows: The user needs to first specify the unique identifiers of the researcher of interest for at least one of ORCID, Google Scholar and Publons. Additionally, a search query for Pubmed can be generated. Furthermore, the option to search for bibliometrics, obtained from the NIH Open Citation Collection using iCite, ^17^ can be selected. After confirmation, publications are retrieved from the specified sources and combined into a table based on the DOI (see Figure 1) or, in case of publications from Google Scholar, based on (fuzzy) matching of titles and/or metadata retrieval from Zotero (Zotero translator, i.e., web scraping) ^18^ or Crossref (i.e., query the available metadata to obtain a DOI). ^19^ Since the set of considered publications stem from the same author, matching of publications is solely based on the title of the publication, by calculating the pairwise relative Levenshtein distances (Levenshtein distance divided by the maximum possible Levenshtein distance, i.e., number of characters) between titles and setting a threshold of 0.1 below which publications are assumed to be the same. No formal validation of this approach was done, but manual checking of a large number cases showed good matching in most cases. After joining the publications list, the open access status of each publication with a DOI is retrieved using Unpaywall, ^20^ who provide a publicly accessible database containing open access information for publications. The definitions of the different open access status that Unpaywall uses is provided in Table 1. Additionally, preprints are defined as having OA status “green” in Unpaywall with the attribute “version” equal to “submittedVersion”. A database snapshot of Unpaywall can be downloaded https://unpaywall.org/products/snapshot.

**Table 1.** Open access (OA) definition used by Unpaywall.

OA status	Open accessible	Description
Gold	Yes	Published in open-access journal
Green	Yes	Publication in free repository
Hybrid	Yes	Open licence
Bronze	Yes	No open licence
Closed	No	

After this step, interactive exploration of the publications is possible. Various options to filter the data according to OA status, year and source (ORCID, Google Scholar, etc.) are available with the possibility to remove or show duplicates (detected using fuzzy matching of titles, similar to matching of publications). Several metrics, tables and plots are available for exploration of the data. Examples include a upset plot that shows how many publications are associated with each identifier, a histogram of the number of publications per year colored by open access status, and a table listing the individual publications. After exploration, specific subsets can be generated using the filtering options, which are then imposed on the visualizations and tables presented. In all cases, relevant snapshots of the citation information can be obtained in the form of a downloadable file.

Another possible application is the integration of local databases, such as university repositories. For example, the Zurich Open Research Archive (ZORA), ^21^ developed and maintained by the Main Library at the University of Zurich, has been integrated in an alternative version of the app that allows local entries to be compared with public profiles, allowing synchronization of publication profiles with local repositories.

### Implementation

The application is written in R (Version 4.1.0) ^22^ and shiny (Version 1.6.0), ^23^ see *Software availability.* As a back-end database, PostgreSQL is used to store a local copy of Unpaywall (and ZORA). Such a local database for Unpaywall is not strictly needed, but a large speedup of the retrieval of the open access status is achieved compared to access over the Unpaywall API. Furthermore, since only a fraction of the data from Unpaywall is used (only the DOI, the open access status and two additional columns for preprint identification) the actual table, containing open access status, is comparably small with a size of about 6 GB (compared to more than 165 GB of the complete version). Unpaywall does daily updates that can be downloaded and are used to update the local database to keep it in sync with the online version. The DOIs for publications listed in Google Scholar are obtained by either matches to publications from other sources, metadata retrieval using the Zotero translator service or a Crossref query.

Various R packages that facilitate retrieval of publications from a specific resource such as https://docs.ropensci.org/rorcid (ORCID), ^24^ https://github.com/jkeirstead/scholar (Google Scholar) ^25^ or https://docs.ropensci.org/rentrez (Pubmed) ^26^ have been included.

### Operation

The app is containerized using Docker (Version 19.03.13, dockerfiles and docker-compose file are provided in *Software availability*). Multiple, interacting containers are deployed using docker-compose, the two most important are a container running the R shiny application and another running PostgreSQL. Furthermore, the Zotero translator service is run in a separate container. As already stated, the PostgreSQL service is not strictly needed, but substantially increases retrieval speed of the OA status.

## Use case

**Figure 2.**
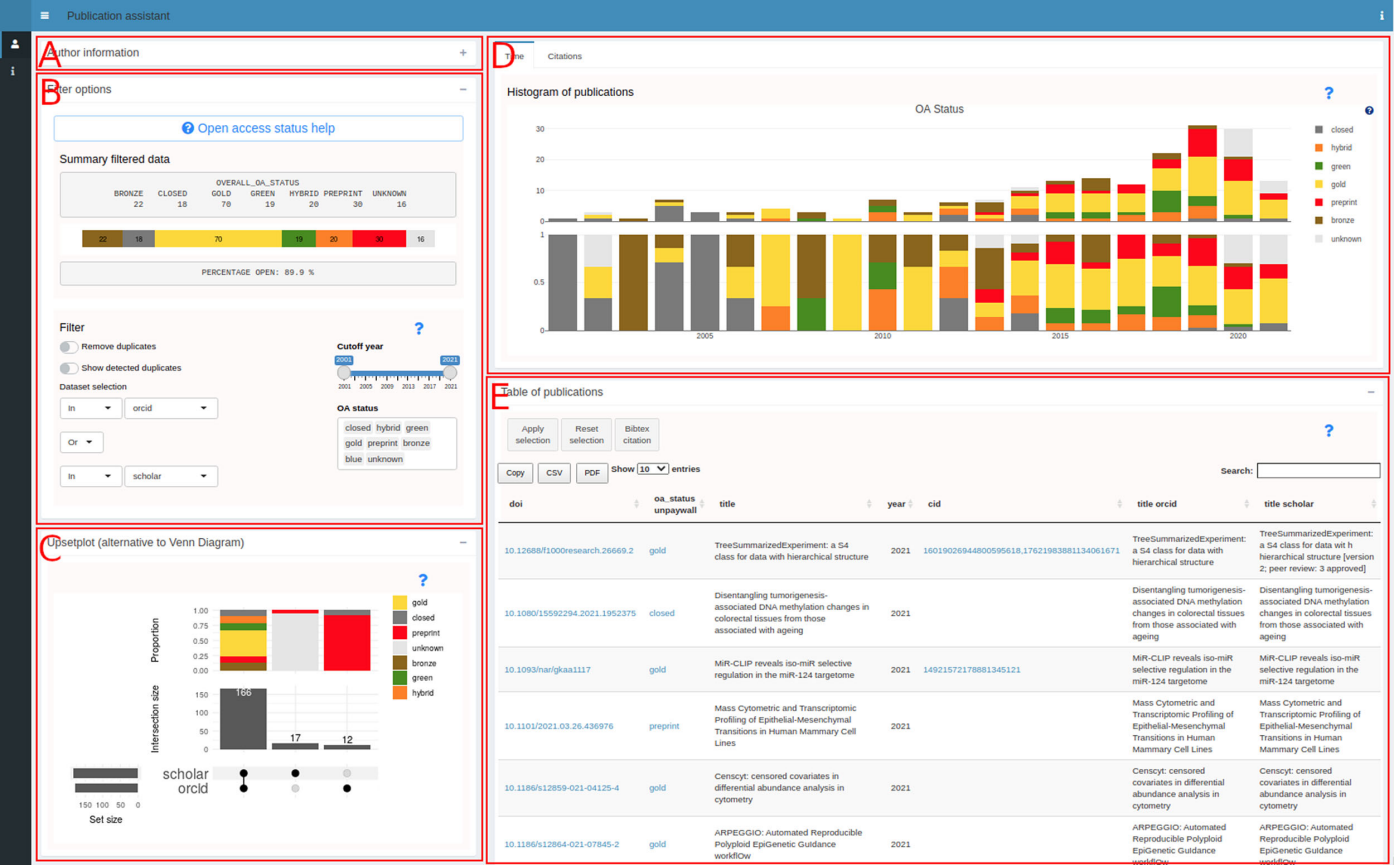
pubassistant.ch panel overview. After entering identifiers in panel A, successful retrieval and merging, panels B-E appear. Panel B is the main panel for filtering. Visualizations are in panel C (upsetplot), D (histogram) and E (table).

Figure 2 shows a use case for an author where the ORCID (0000-0002-3048-5518) and Google Scholar ID (XPfrRQEAAAAJ) were given as an input (collapsed panel in Figure 2A). Panel B provides a summary of the publication list and options to filter by dataset, by year and by OA status. Additionally, the possibility to remove duplicates or only show duplicates is available. The other panels contain visualizations including an upsetplot ^27^ (C), a histogram (D) and a table (E). The table can be further filtered by selecting rows allowing to create specific citation lists that can be created based on the rows in the table. The contents of the table can be copied to the clipboard or downloaded in CSV format.

## Discussion

Our method relies on the DOI to retrieve the OA status, which is a limitation in domains where DOIs are not used. The DOI is also used to unambiguously match publications. If no DOI is present, the titles of the publications are used for matching, which can lead to ambiguity. Even if a publication has an assigned DOI, but it is missing in the data, it becomes difficult or time-consuming to retrieve the missing information with services such as the Zotero translator or Crossref.

Because of the non commercial nature of this application, some additional limits present themselves. Most notably, our application requires freely-available APIs, or in the case of Google Scholar web-scraping (contravening the terms of use of Google Scholar), for retrieving the open publication data from their respective sources. For the two main sources considered so far (ORCID and Google Scholar), no restrictions have been noticed, while for others rate limits in the number of requests are quite restrictive (e.g., for Publons). Other APIs not currently included in our application (e.g., from Dimensions or Mendeley) could be added in the future.

## Data availability

No data are associated with this article.

## Software availability

Software available from: https://pubassistant.ch/

Source code available from: https://github.com/markrobinsonuzh/os_monitor

Archived source code at time of publication: https://doi.org/10.5281/zenodo.5509626

License: MIT

## References

[1] UNESCO: Science Report. 2021. Reference Source

[2] Bornmann L Mutz R : Growth rates of modern science: A bibliometric analysis based on the number of publications and cited references. *J. Assoc. Inf. Sci. Technol.* 2015;66(11):2215–2222. 2330-1643. 10.1002/asi.23329 Reference Source Reference Source

[3] Haak LL Fenner M Paglione L : *ORCID: a system to uniquely identify researchers.* Learned Publishing;2012;25(4):259–264. 1741-4857. 10.1087/20120404 Reference Source Reference Source

[4] Google Scholar. Reference Source

[5] Publons. Reference Source

[6] Hook DW Porter SJ Herzog C : Dimensions: Building Context for Search and Evaluation. *Front. Res. Met. Analy.* 23, August 2018;3: 2504-0537. 10.3389/frma.2018.00023 Reference Source

[7] World’s largest publisher-neutral citation index and research intelligence platform. Reference Source

[8] Vale RD : Accelerating scientific publication in biology. *Proc. Natl. Acad. Sci.* National Academy of Sciences Section: Perspective;November 2015;112(44):13439–13446. 0027-8424, 1091-6490. 10.1073/pnas.1511912112 Reference Source 26508643 PMC4640799

[9] Johansson MA Reich NG Meyers LA : Preprints: An underutilized mechanism to accelerate outbreak science. *PLoS Med.* Public Library of Science;April 2018;15(4):e1002549. 1549-1676. 10.1371/journal.pmed.1002549 29614073 PMC5882117

[10] Open Access. Reference Source

[11] Conlon M Woods A Triggs G : VIVO: a system for research discovery. *J. Open Source Softw.* 2019;4:1182. 10.21105/joss.01182

[12] *Deployments · wcmc-its/ReCiter* : (kein Datum). Abgerufen am 15. 11 2021 von ReCiter: an enterprise open source author disambiguation system for academic institutions. Reference Source

[13] *Impactstory: Discover the online impact of your research* : (kein Datum). Abgerufen am 15. 11 2021 von. Reference Source

[14] *Profiles Research Networking Software* : (kein Datum). Abgerufen am 15. 11 2021 von. Reference Source

[15] Trends for open access to publications. Reference Source

[16] SNSF Open Access Check. Reference Source

[17] ICite, B: Ian Hutchins, and George Santangelo. iCite Database Snapshots (NIH Open Citation Collection). The NIH Figshare Archive;2019. 10.35092/YHJC.C.4586573 Reference Source

[18] Zotero Translation Server:July 2021. original-date: 2018-06-11T11:28:53Z. Reference Source

[19] Lammey R : CrossRef developments and initiatives: an update on services for the scholarly publishing community from CrossRef.page6.

[20] Piwowar H Priem J Larivière V : The state of OA: a large-scale analysis of the prevalence and impact of Open Access articles. *PeerJ.* February 2018;6:e4375. 2167-8359. 10.7717/peerj.4375 29456894 PMC5815332

[21] Welcome to Zurich Open Repository and Archive - Zurich Open Repository and Archive. Reference Source

[22] R R Development Core Team: *R: A Language and Environment for Statistical Computing.* R Foundation for Statistical Computing;2011; 3-900051-07-0. 16000706. 10.1007/978-3-540-74686-7

[23] Chang W Cheng J Allaire JJ : shiny: Web Application Framework for R. 2020. Reference Source

[24] Scott Chamberlain: rorcid: Interface to the ‘Orcid.org’ API. 2021.

[25] Keirstead J : scholar: analyse citation data from Google Scholar. 2016. Reference Source

[26] Winter DJ : rentrez: an R package for the NCBI eUtils API. *The R Journal.* 2017;9(2):520–526. 10.32614/RJ-2017-058

[27] Conway JR Lex A Gehlenborg N : UpSetR: an R package for the visualization of intersecting sets and their properties. *Bioinformatics.* September 2017;33(18):2938–2940. 1367-4811. 10.1093/bioinformatics/btx364 28645171 PMC5870712

