# Supplementary material for: pubassistant.ch: consolidating publication profiles of researchers
Source: F1000Res. 2022 Apr 13;10:989. Originally published 2021 Sep 30. [Version 3] doi: 10.12688/f1000research.73493.3 (PMC9010799; doi:10.12688/f1000research.73493.3)
Supplement: Peer review history

# Peer review: Reviewer response for version 3

**DOI:** 10.5256/f1000research.126603.r134743

**Published:** 2022-05-04

**Authors:** Curtis L. Cole, Paul Albert

**Affiliations:** 1 Wood Library, Weill Cornell Medical College, New York, NY, USA; 2 Department of Population Health Sciences, Weill Cornell Medical College, New York, NY, USA

The authors did address several of our concerns and clearly disagree with much of our feedback. Some of the improvements they made are not reflected in the figures (e.g. Fig 2) so with little effort they could make more improvements. But we agree with the other reviewer's observation that the code itself might be useful and should be shared.

Are the conclusions about the tool and its performance adequately supported by the findings presented in the article?

No

Is the rationale for developing the new software tool clearly explained?

No

Is the description of the software tool technically sound?

Yes

Are sufficient details of the code, methods and analysis (if applicable) provided to allow replication of the software development and its use by others?

Yes

Is sufficient information provided to allow interpretation of the expected output datasets and any results generated using the tool?

No

Reviewer Expertise:

We have both collaborated on publication tracking and disambiguation systems, such as VIVO and Reciter.

We confirm that we have read this submission and believe that we have an appropriate level of expertise to confirm that it is of an acceptable scientific standard.

# Peer review: Reviewer response for version 3

**DOI:** 10.5256/f1000research.126603.r134741

**Published:** 2022-04-14

**Authors:** Daniel W Hook

**Affiliations:** 1 Digital Science, London, UK

I thank the authors for making further updates in response to my comments. In light of their diligent changes, I am pleased to recommend this article to others.

Are the conclusions about the tool and its performance adequately supported by the findings presented in the article?

No

Is the rationale for developing the new software tool clearly explained?

No

Is the description of the software tool technically sound?

Partly

Are sufficient details of the code, methods and analysis (if applicable) provided to allow replication of the software development and its use by others?

Yes

Is sufficient information provided to allow interpretation of the expected output datasets and any results generated using the tool?

No

Reviewer Expertise:

Open Research, Bibliometrics, Sociology of Research, Theoretical Physics (Quantum Statistical Mechanics, PT-Symmetric Quantum Mechanics).

I confirm that I have read this submission and believe that I have an appropriate level of expertise to confirm that it is of an acceptable scientific standard.

# Peer review: Reviewer response for version 2

**DOI:** 10.5256/f1000research.80816.r120551

**Published:** 2022-02-17

**Authors:** Griffin M Weber

**Affiliations:** 1 Department of Biomedical Informatics, Harvard Medical School, Boston, MA, USA; 2 Department of Medicine, Beth Israel Deaconess Medical Center, Boston, MA, USA

pubassistant.ch is a website that helps users to manage the list of publications that they have authored. Publications can be imported from several sources, including ORCID and Google Scholar. Deduplication is handled by matching on DOI or similar titles (using Levenshtein distance). The software automatically imports metrics on the publications, such as their open access status and number of citations. Various visualizations show summary information about the publications. The publication list can be exported in several formats.

 There are other software programs that provide similar functionality: reference management, open access reporting, and bibliometric analyses. Though, as the authors correctly point out, many of these are products that institutions acquire and make available only to their community. These include Dimensions, Web of Science, VIVO, and Profiles RNS. However, OpenVIVO and Mendeley allow anyone to sign up. Also, users of ORCID can import publications from Scopus.

 The individual components of pubassistant.ch are not novel: the authors use public APIs and well known data sources, and their deduplication process uses a common algorithm. However, when put together, the website presents the data in a nice way. I liked the timelines and UpSet plot showing the different open access levels of the publications. It was easy to interpret the visualizations and understand how to use their settings.

 Unfortunately, some features of the website are slow due to limits on the APIs the software calls; and, I encountered some bugs, especially with the Citations tab either not loading or producing an error. The reliance on web-scraping Google Scholar is problematic. It should be noted that publications from Google Scholar can be exported in BibTeX format and then imported into ORCID, which is alternative way of merging Google Scholar and ORCID publication lists, though pubassistant.ch

 I’m not sure who the users of this website will be. Investigators managing their ORCID and Google Scholar profiles might not like having to go to a third website. Funding agencies, librarians, and institution administrators would probably want the open access and citation metrics, but having to enter IDs manually for each author one at a time and waiting for the results could be too time consuming.

 I think the biggest impact of pubassistant.ch could be the code itself. The website is a great demonstration of how to (1) query the APIs some frequently used bibliometric tools, (2) integrate the data that are returned, and (3) visualize the results in some interesting ways. I can imagine this code being extended by other developers or even embedded in other software platforms in the future.

Are the conclusions about the tool and its performance adequately supported by the findings presented in the article?

Yes

Is the rationale for developing the new software tool clearly explained?

Yes

Is the description of the software tool technically sound?

Yes

Are sufficient details of the code, methods and analysis (if applicable) provided to allow replication of the software development and its use by others?

Yes

Is sufficient information provided to allow interpretation of the expected output datasets and any results generated using the tool?

Yes

Reviewer Expertise:

bibliometric analysis, social network analysis, research networking systems

I confirm that I have read this submission and believe that I have an appropriate level of expertise to confirm that it is of an acceptable scientific standard.

# Response

**Published:** 2022-04-11

**Authors:** Reto Gerber

**Affiliations:** University of Zurich, Switzerland

*Unfortunately, some features of the website are slow due to limits on the APIs the software calls; and, I encountered some bugs, especially with the Citations tab either not loading or producing an error.*

- **Some of the less stable components of the applications have been deactivated. **

*The reliance on web-scraping Google Scholar is problematic. It should be noted that publications from Google Scholar can be exported in BibTeX format and then imported into ORCID, which is alternative way of merging Google Scholar and ORCID publication lists, though pubassistant.ch*

**

*I’m not sure who the users of this website will be. Investigators managing their ORCID and Google Scholar profiles might not like having to go to a third website. Funding agencies, librarians, and institution administrators would probably want the open access and citation metrics, but having to enter IDs manually for each author one at a time and waiting for the results could be too time consuming.*

- **The target user could be a researcher who wants to align their multiple public profiles in a systematic and semi-automatic way that also gives information about timelines and open access. The manuscript has been updated to make it clearer who the target user is.**

# Peer review: Reviewer response for version 2

**DOI:** 10.5256/f1000research.80816.r120552

**Published:** 2022-01-31

**Authors:** Curtis L. Cole, Paul Albert

**Affiliations:** 1 Wood Library, Weill Cornell Medical College, New York, NY, USA; 2 Department of Population Health Sciences, Weill Cornell Medical College, New York, NY, USA

This paper describes pubassistant.ch, a freely available website using open-source code. Pubassistant.ch is designed to perform the following functions:

- Allows users to manually import data from the following services: Google Scholar, Publons, PubMed, and ORCID.
- Disambiguate (deduplicate) articles.
- Provide the open access status of each article.
- Allow users to download citation citation data as Bibtex for updating these profiles.

We applaud the initiative to design and deploy a tool meant to improve researchers' work lives.

 We agree with the first reviewer's careful comments and appreciate that many of the concerns were addressed in this revision. However, one key problem remains, the unclear perspective and motivation for the tool. A key question for any software tool is whether it addresses an unmet need. It's not quite clear what this tool could uniquely perform. Is it that Pubassistant allows users to easily sync publication lists between profile systems? Maybe the tool provides readily available statistics on open access status of a given author's output. “Who exactly is the target user?” I don’t think the author would agree this tool is designed for unaffiliated researchers.

 While this revision does now mention many other contributions in this area, it remains unclear what niche pubassistant.ch is filling within this larger space. Also, as noted by the first reviewer, Vivo should be written VIVO.

 While any software, especially open source, is often used in ways not imagined by the original author, without a key use case the success or failure of the program to meet its goal cannot be evaluated. This points to one of the key problems with the paper which is the lack of any evaluation other than a high level subjective sense that it works.

 In our opinion, the functionality in pubassistant.ch is not ready for deployment calling into question even that subjective assessment. It has several bugs and could benefit from user feedback and iteration. For example:

- This reviewer could not perform a PubMed search until clicking on the "Example PubMed Query" button.
- The "Time" tab displays two histograms. The top graph would appear to be a raw count and the bottom graph is a percentage by year. But this distinction is not explained by the site either via a text-based description or using any labels on the axes. In this reviewer's estimation, only one graph is warranted.
- The units on the "Citations" graph are sometimes unnecessarily unconventional. One year is "2,016.5". Years do not typically include commas or periods which makes the display confusing.
- The x-axis dropdown in the "Citations" tab shows up behind the "Copy", "CSV", and "Excel" buttons.
-  After attempting to click on the "Download Bibtex citation" button, this user got a "503 Service Unavailable" error. This did not allow this reviewer to verify whether the tool can allow users to populate publication lists into a tool like Google Scholar.
- The integration with Sci-Hub has a certain illicit appeal. But when clicking on the "Get Full text links" button, nothing happens.
- The "Apply selection" button is confusing. One assumes this applies to the filter in the left hand column. Wouldn't this button be more appropriate to include immediately below the filters?
- When this reviewer clicks on "Remove duplicates" and then "Apply selection," it removes all data from the table including non-duplicates.
-  In the "Table of publications" section, the label "cid" is used. What does this label mean? One assumes it's a unique ID that Google uses. A more descriptive label might be more appropriate. 
- The method for checking for duplicates is to look for duplicate article titles within the same author's namespace.
- As designed, the article disambiguation can run into problems when there are no DOIs. This is a concern in cases where publication records are imported from Google Scholar as Pubassistant.ch appears to only provide article title and Google Scholar's local identifier. The authors indicate that they use fuzzy matching on article title with an author's namespace to perform deduplication. In the context of a research article, such an assertion should be tested and validated. Here's an example where such an approach would fail. All the following articles were written by Robert S. Brown, a faculty member at Weill Cornell Medicine, and all of them have article titles that are non-unique within Robert Brown's namespace: See here.

This is an interesting work that has the potential to be a real contribution, but some additional development is probably warranted before publication and distribution.

Are the conclusions about the tool and its performance adequately supported by the findings presented in the article?

No

Is the rationale for developing the new software tool clearly explained?

No

Is the description of the software tool technically sound?

Yes

Are sufficient details of the code, methods and analysis (if applicable) provided to allow replication of the software development and its use by others?

Yes

Is sufficient information provided to allow interpretation of the expected output datasets and any results generated using the tool?

No

Reviewer Expertise:

We have both collaborated on publication tracking and disambiguation systems, such as VIVO and Reciter.

We confirm that we have read this submission and believe that we have an appropriate level of expertise to state that we do not consider it to be of an acceptable scientific standard, for reasons outlined above.

# Response

**Published:** 2022-04-11

**Authors:** Reto Gerber

**Affiliations:** University of Zurich, Switzerland

*We agree with the first reviewer's careful comments and appreciate that many of the concerns were addressed in this revision. However, one key problem remains, the unclear perspective and motivation for the tool. A key question for any software tool is whether it addresses an unmet need. It's not quite clear what this tool could uniquely perform. Is it that Pubassistant allows users to easily sync publication lists between profile systems? Maybe the tool provides readily available statistics on open access status of a given author's output. “Who exactly is the target user?” I don’t think the author would agree this tool is designed for unaffiliated researchers.*

- **The target user could be a researcher who wants to align their multiple public profiles in a systematic and semi-automatic way that also gives information about timelines and open access. The manuscript has been updated to make it clearer who the target user is.**

*** ***


*While this revision does now mention many other contributions in this area, it remains unclear what niche pubassistant.ch is filling within this larger space. Also, as noted by the first reviewer, Vivo should be written VIVO.*

*While any software, especially open source, is often used in ways not imagined by the original author, without a key use case the success or failure of the program to meet its goal cannot be evaluated. This points to one of the key problems with the paper which is the lack of any evaluation other than a high level subjective sense that it works.*

- **Pubassistant.ch relies heavily on other tools to interact with the various APIs (R packages, such as rorcid), retrieve open access information (Unpaywall), and retrieval of DOI from metadata (Crossref, Zotero translator). We assume that those heavily used tools work as intended. The main part of the applications that need evaluation in our opinion is the matching of publications. A note on the accuracy of detecting duplicates using a small dataset consisting of publications with associated preprints was added. **

** **


*In our opinion, the functionality in pubassistant.ch is not ready for deployment calling into question even that subjective assessment. It has several bugs and could benefit from user feedback and iteration. For example:*

- *This reviewer could not perform a PubMed search until clicking on the "Example PubMed Query" button.*
  - **Added placeholder text to indicate that the button on the right has to be pressed first.**
**  **
- *The "Time" tab displays two histograms. The top graph would appear to be a raw count and the bottom graph is a percentage by year. But this distinction is not explained by the site either via a text-based description or using any labels on the axes. In this reviewer's estimation, only one graph is warranted.*
  - **The missing axis labels have been added.**
**  **
- *The units on the "Citations" graph are sometimes unnecessarily unconventional. One year is "2,016.5". Years do not typically include commas or periods which makes the display confusing.*
  - **The experimental “Citations” tab has been deactivated.**
**  **
- *The x-axis dropdown in the "Citations" tab shows up behind the "Copy", "CSV", and "Excel" buttons.*
  - **The experimental “Citations” tab has been deactivated.**
**  **
- * After attempting to click on the "Download Bibtex citation" button, this user got a "503 Service Unavailable" error. This did not allow this reviewer to verify whether the tool can allow users to populate publication lists into a tool like Google Scholar.*
  - **This bug has been fixed.**
**  **
- *The integration with Sci-Hub has a certain illicit appeal. But when clicking on the "Get Full text links" button, nothing happens.*
  - **This feature has been deactivated.**
**  **
- *The "Apply selection" button is confusing. One assumes this applies to the filter in the left hand column. Wouldn't this button be more appropriate to include immediately below the filters?*
  - **The “Apply selection” button does filter entries selected in the list below. The name of the button has been changed and additional hints were added to make this clearer.**
**  **
- *When this reviewer clicks on "Remove duplicates" and then "Apply selection," it removes all data from the table including non-duplicates.*
  - **This behavior is as intended if no rows in the table have been selected. The same will happen without pressing “Remove duplicates” or with pressing “Show detected duplicates”.**
**  **
- * In the "Table of publications" section, the label "cid" is used. What does this label mean? One assumes it's a unique ID that Google uses. A more descriptive label might be more appropriate. *
  - **“Cid” is an identifier for publications used by Google. This label has been extended to make it clearer.**
**  **

*As designed, the article disambiguation can run into problems when there are no DOIs. This is a concern in cases where publication records are imported from Google Scholar as Pubassistant.ch appears to only provide article title and Google Scholar's local identifier. The authors indicate that they use fuzzy matching on article title with an author's namespace to perform deduplication. In the context of a research article, such an assertion should be tested and validated. Here's an example where such an approach would fail. All the following articles were written by Robert S. Brown, a faculty member at Weill Cornell Medicine, and all of them have article titles that are non-unique within Robert Brown's namespace: See here.*

- **It is true that the finding of duplicates with only the title will not always work and with the available data (not all available data is shown in the table), such as coauthors, publication date, open access status, etc., there are certainly improvements possible. The duplication identification performed in Pubassistant.ch is mainly intended to assist in finding duplicates and manual checking of results can be easily done because of the interactivity provided in the app. **

# Peer review: Reviewer response for version 2

**DOI:** 10.5256/f1000research.80816.r116162

**Published:** 2022-01-12

**Authors:** Daniel W Hook

**Affiliations:** 1 Digital Science, London, UK

We thank the authors for taking the time to improve their manuscript.

 We make a number of optional suggestions for changes below that we believe would further improve and strengthen the paper:

1. The authors note that “Many tools, both commercial and free, exist to explore certain aspects of bibliographies”.  While this is true it does not, perhaps, get at the core of what is intended.  We suggest that the authors consider revision of this comment.  We suggest that a text similar to the following may be appropriate: “Many tools, both commercial and free, exist to bring data bibliographic and bibliometric data together to meet a multitude of different use cases including: evaluation, compliance (e.g. OA), grant writing, literature review and keeping professional web profiles updated.”
2. The authors go on to state: “Some of the existing commercial tools including Elements (from the company Symplectic) and Dimensions, both of which are mainly intended for institutional use; but, especially Dimensions also offers functionalities for authors to explore their bibliographies”.  We suggest that the authors consider revision along the lines of: “There is a distinct segment of research analysis and compliance tools that provides functionality to research institutions to allow research profiles to be curated to meet some, but not all, of the aforementioned use cases.  Notably, these systems do not appear to have been designed (nor are individual subscriptions or versions available) to meet the needs of individuals who wish to curate their own individual profiles outside an institutional context.  A key use case that has emerged for contemporary research is to have a detailed understanding of their bibliometric rather than just their bibliographic profile.  Given the growth of availability of bibliometric information and the growing need to use bibliometric information as a tool for academic decision making in recent years, the lack of software to bring this information together for the individual appears to be an omission in the tools landscape.”  It may also be helpful to note that tools such as EndNote, Papers and others also appear not to meet this need. 
  We believe that the authors should be more forthright in identifying the key use cases for their tool as this strengthens the rationale for their software.
3. The authors observe that “But since it is possible that publications listed in Google Scholar are not in ORCID, the reverse needs to be done to be sure the accounts are up to date. If more accounts need to be synced (e.g., Publons), the complexity and time needed increases accordingly. Although it is possible, and probably advisable, to link accounts for automatic updates (e.g., linking Publons with ORCID), this cannot be done under all circumstances and missing publications are still possible.”  We suggest that it would be helpful to mention that most CRIS systems now include the ability to upload affiliation information to ORCID records from institutional systems; that Crossref/ORCID auto-update functionality (https://support.orcid.org/hc/en-us/articles/360006971293-Auto-updates-in-third-party-systems-Crossref) and that the ability to link ORCID to a Dimensions account (https://www.dimensions.ai/blog/digital-science-announces-dimensions-integration-of-orcid/) are all possible routes to updating data in ORCID.  In addition, ORCID has released tools to ensure that full provenance is captured in these updates (https://info.orcid.org/documentation/api-tutorials/api-tutorial-add-and-update-data-on-an-orcid-record/#easy-faq-2692).
4. We believe that Vivo should be VIVO.
5. The authors may wish to consider mentioning that the new API from OpenAlex (https://openalex.org/) could be a useful addition to their data sources by providing improved article metadata and citation information within an appropriate data licensing context.
6. It would be healthy and helpful for the context of the article and of the software to include mention of DORA, the Leiden Manifesto and the responsible research evaluation movement in the motivation for the software.  Bibliometrics can be interesting and informative but do not provide a comprehensive overview of an academic.

Are the conclusions about the tool and its performance adequately supported by the findings presented in the article?

No

Is the rationale for developing the new software tool clearly explained?

No

Is the description of the software tool technically sound?

Partly

Are sufficient details of the code, methods and analysis (if applicable) provided to allow replication of the software development and its use by others?

Yes

Is sufficient information provided to allow interpretation of the expected output datasets and any results generated using the tool?

No

Reviewer Expertise:

Open Research, Bibliometrics, Sociology of Research, Theoretical Physics (Quantum Statistical Mechanics, PT-Symmetric Quantum Mechanics).

I confirm that I have read this submission and believe that I have an appropriate level of expertise to confirm that it is of an acceptable scientific standard, however I have significant reservations, as outlined above.

# Response

**Published:** 2022-04-11

**Authors:** Reto Gerber

**Affiliations:** University of Zurich, Switzerland

*The authors note that “Many tools, both commercial and free, exist to explore certain aspects of bibliographies”.  While this is true it does not, perhaps, get at the core of what is intended.  We suggest that the authors consider revision of this comment.  We suggest that a text similar to the following may be appropriate: “Many tools, both commercial and free, exist to bring data bibliographic and bibliometric data together to meet a multitude of different use cases including: evaluation, compliance (e.g. OA), grant writing, literature review and keeping professional web profiles updated.”*

- **This part of the text has now been updated to be more specific.**
**  **

*The authors go on to state: “Some of the existing commercial tools including Elements (from the company Symplectic) and Dimensions, both of which are mainly intended for institutional use; but, especially Dimensions also offers functionalities for authors to explore their bibliographies”.  We suggest that the authors consider revision along the lines of: “There is a distinct segment of research analysis and compliance tools that provides functionality to research institutions to allow research profiles to be curated to meet some, but not all, of the aforementioned use cases.  Notably, these systems do not appear to have been designed (nor are individual subscriptions or versions available) to meet the needs of individuals who wish to curate their own individual profiles outside an institutional context.  A key use case that has emerged for contemporary research is to have a detailed understanding of their bibliometric rather than just their bibliographic profile.  Given the growth of availability of bibliometric information and the growing need to use bibliometric information as a tool for academic decision making in recent years, the lack of software to bring this information together for the individual appears to be an omission in the tools landscape.”  It may also be helpful to note that tools such as EndNote, Papers and others also appear not to meet this need. *

- **This part of the text has also been updated to be more specific.**

*We believe that the authors should be more forthright in identifying the key use cases for their tool as this strengthens the rationale for their software.*

- **One specific use case of pubassistant is to find publications in one public profile that are missing in another profile. This frequently happens when, for instance, the ORCID is missing (e.g., a collaborator forgets to add yours) and thus the public profile of ORCID will not show the respective publication although it might be recognized (automatically) by Google Scholar. A clearer description of the key use case has now been added to the manuscript.**
**  **

*The authors observe that “But since it is possible that publications listed in Google Scholar are not in ORCID, the reverse needs to be done to be sure the accounts are up to date. If more accounts need to be synced (e.g., Publons), the complexity and time needed increases accordingly. Although it is possible, and probably advisable, to link accounts for automatic updates (e.g., linking Publons with ORCID), this cannot be done under all circumstances and missing publications are still possible.”  We suggest that it would be helpful to mention that most CRIS systems now include the ability to upload affiliation information to ORCID records from institutional systems; that Crossref/ORCID auto-update functionality (https://support.orcid.org/hc/en-us/articles/360006971293-Auto-updates-in-third-party-systems-Crossref) and that the ability to link ORCID to a Dimensions account (https://www.dimensions.ai/blog/digital-science-announces-dimensions-integration-of-orcid/) are all possible routes to updating data in ORCID.  In addition, ORCID has released tools to ensure that full provenance is captured in these updates ( https://info.orcid.org/documentation/api-tutorials/api-tutorial-add-and-update-data-on-an-orcid-record/#easy-faq-2692).*

- **The set of possible methods to automatically update data in ORCID has now been added to the manuscript.**
**  **

*We believe that Vivo should be VIVO.*

- **Vivo has been renamed to VIVO in the manuscript.**
**  **

*The authors may wish to consider mentioning that the new API from OpenAlex (https://openalex.org/) could be a useful addition to their data sources by providing improved article metadata and citation information within an appropriate data licensing context.*

- **Possible extension with OpenAlex has been added to the discussion.**
**  **

*It would be healthy and helpful for the context of the article and of the software to include mention of DORA, the Leiden Manifesto and the responsible research evaluation movement in the motivation for the software.  Bibliometrics can be interesting and informative but do not provide a comprehensive overview of an academic.*

- **A note about DORA and the Leiden manifesto has now been added to the introduction.**

# Peer review: Reviewer response for version 1

**DOI:** 10.5256/f1000research.77148.r96152

**Published:** 2021-10-26

**Authors:** Daniel W Hook

**Affiliations:** 1 Digital Science, London, UK

The authors have created a free, open source piece of software to bring researcher and publication records together from different data sources. They detail their motivations and methodology in this paper.

- The authors begin their article by motivating the development of their software based on the difficulty of consolidating all the publications from a single author resulting from:
   - The non-uniqueness of names of authors
   - The proliferation of unique identifiers that aim to solve this problem (e.g. ResearcherID/Publons, ORCID *et al.*)It is reasonable to claim that we, as a community, have not yet realised a comprehensive solution to these problems. 
  However, the authors choose to take a peculiarly western-centric view of this issue. The greatest challenges of name disambiguation are typically found when authors who might not natively use a roman alphabetic system are forced to transliterate their names when publishing in the western-centred publishing system. This fact goes unacknowledged in this paper but would seem to be at the heart of the name disambiguation issue.
  There are several approaches to name disambiguation but there are broadly two “schools”: attended and unattended. “Attended” is where humans interact with the disambiguation and “unattended” is where humans have no role. Attended disambiguation is the focus for identity management systems like ORCID - incentives need to be aligned for authors and others to participate in this and significant work goes into understanding the motivations and concerns of researchers in order to make these types of system successful. Unattended disambiguation makes use of the data that are available in the ecosystem (including the outputs of the attended disambiguation approach) to create a calculated output. We feel that the authors should give some of this background in their paper for context.
  In this context, it is important to acknowledge that only one identifier system has been successful in engaging the broad global academic community and that is ORCID. Other solutions are self-acknowledged proprietary solutions that aim to solve this problem in limited contexts (Researcherid.com/Publons to improve the Web of Science data (attended disambiguation); ScopusID - unattended disambiguation approach). Dimensions explicitly leverage ORCID data in an unattended person disambiguation approach. ^1^
- The authors claim that there is no standardization of unique identifiers for authors and documents.
  This is a very strong claim when viewed at an international ecosystem level. The majority of funders, publishers, institutions, scholarly societies and government agencies involved with research from around the world acknowledge DOIs as the key identifier for a research paper and ORCID as the principal identifier of researchers. 
  Some complexity lies in the authority that issues and maintains an article DOI (Crossref or DataCite in most cases but also, for example, J-Stage in Japan). 
  ORCID may currently be a “de facto standard” but the authors’ statement to this effect underplays the central role played by ORCID in the scholarly infrastructure community and the extent to which ORCID is the standard with which the majority of the commercial and open infrastructure providers engage. Google Scholar provides no API and makes no claim of persistence of identifiers; authors have limited control of their profile and of privacy. Researcherid.com (the progenitor of Publons/ResearcherID) is a founding member, supporter and participant of ORCID. We suggest that the authors should ensure that acknowledgement be made of ORCID’s suitability and level of adoption in academia. If the authors wish to note that ORCID is not the only standard, it would be appropriate to mention parallel efforts such as researchmap.jp in Japan and note that some countries remain reluctant to adopt ORCID as their principal identifier at this time.
- The authors claim that it is not easy to determine the provenance of data in Dimensions has derived its data from ORCID or Publons. Arguably this is not the case. Dimensions clearly states that algorithmic methods are used with the input of ORCID data. ^1^ Publons/Researchid.com data is a proprietary source that could not be used in Dimensions without explicit acknowledgement of its use. 
- The authors further claim that no information is given about the completeness of these sources (e.g. Dimensions/Web of Science/Scopus), yet significant academic work has been undertaken to understand and benchmark coverage and completeness of these sources (see reference 2 for example). ^2^
- We agree with the authors that Open Science is indeed critical. This is why Dimensions, Web of Science, Scopus and others contain information on Open Access statuses of articles, provided by Unpaywall ( https://unpaywall.org/integrations). Dimensions also contains full listings of preprint articles from many preprint servers including ArXiv.org, BioArXiv, the Center for Open Science, PeerJ and so on. As such we find the authors’ comment that these things are “not taken into account” to be misleading.
- The authors then appear to contradict themselves by claiming that preprints and publications cause duplicate entries in these systems. In Dimensions, where the current reviewer has the most experience, publications from preprints are linked to final publications where this information is available, see for example https://app.dimensions.ai/details/publication/pub.1118864658?and_facet_researcher=ur.01123321343.51. We agree that manual intervention is often needed in these cases.
- The authors claim that a free tool does not exist to explore metadata brought together from multiple sources, however, this does not acknowledge the rich lineage of free tools written to serve institutional use cases in author disambiguation and metadata aggregation including: VIVO ( https://duraspace.org/vivo/) and Catalyst Profiles ( http://profiles.catalyst.harvard.edu), both funded by the NIH; ImpactStory (e.g. https://profiles.impactstory.org/u/0000-0001-6728-7745/publications); and, ReCiter ( https://github.com/wcmc-its/ReCiter). The authors should endeavour to situate their work in the context of this prior work.
- Symplectic Elements is, as the authors state, a commercial system focused on institutional use cases that meets this need. However, the authors fail to acknowledge that outside STEM subjects, coverage of research outputs becomes more challenging. The strength of commercial tools is often that, as they must meet institutional needs, work is put into diversifying coverage of different output types beyond the journals and conference proceedings that STEM subjects favour. While composing a faithful representation of a STEM academic and their output can be challenging from disparate data sources, doing the same for a non-STEM academic can be an order of magnitude more difficult. We believe that it is also fair to acknowledge that institutional engagement is not unimportant to improving the overall data quality in the bibliographic ecosystem.
- The authors also appear to be unaware that Dimensions does bring records together from multiple sources including author information and that this is transparently documented in scholarly articles written by the Dimensions team, ^1^ as well as in system and API documentation ( https://docs.dimensions.ai/dsl/). Open access information and much more (citation information, funding information) and is available in the version of Dimensions that is available for free for personal use. 
- The authors note that Dimensions, Mendeley and others do not offer a public open API.
  While Dimensions does offer a free end-user tool, it does not offer a full open public API. However, Dimensions does offer a free metrics API for non-commercial purposes ( https://www.dimensions.ai/dimensions-apis/) and Mendeley continues to offer a free API (albeit with recent changes to some of the functionality around search functionality - https://dev.mendeley.com/).
  In addition, given the use of Google Scholar by the authors in their tool, I think that it is important to note explicitly that Google Scholar does not offer an API. Indeed, from the software source code deposited by the authors, it appears that data is scraped from the Google Scholar website contravening the terms of use of Google Scholar. This fact should be explicitly noted in the paper.
- The paper contains no critical analysis on the accuracy or success of the algorithmic approaches taken by the authors as regards fuzzy matching of papers and authors between sources.
- The paper would also be improved by including a flow diagram and description of matching approach taken by the authors.

Are the conclusions about the tool and its performance adequately supported by the findings presented in the article?

No

Is the rationale for developing the new software tool clearly explained?

No

Is the description of the software tool technically sound?

Partly

Are sufficient details of the code, methods and analysis (if applicable) provided to allow replication of the software development and its use by others?

Yes

Is sufficient information provided to allow interpretation of the expected output datasets and any results generated using the tool?

No

Reviewer Expertise:

Open Research, Bibliometrics, Sociology of Research, Theoretical Physics (Quantum Statistical Mechanics, PT-Symmetric Quantum Mechanics).

I confirm that I have read this submission and believe that I have an appropriate level of expertise to state that I do not consider it to be of an acceptable scientific standard, for reasons outlined above.

## References

[1] : Dimensions: Building Context for Search and Evaluation. *Frontiers in Research Metrics and Analytics* .2018;3: 10.3389/frma.2018.00023 10.3389/frma.2018.00023

[2] : Large-scale comparison of bibliographic data sources: Scopus, Web of Science, Dimensions, Crossref, and Microsoft Academic. *Quantitative Science Studies* .2021;2(1) : 10.1162/qss_a_00112 20-41 10.1162/qss_a_00112

# Response

**Published:** 2021-12-10

**Authors:** Reto Gerber

**Affiliations:** University of Zurich, Switzerland

- *However, the authors choose to take a peculiarly western-centric view of this issue. The greatest challenges of name disambiguation are typically found when authors who might not natively use a roman alphabetic system are forced to transliterate their names when publishing in the western-centred publishing system. This fact goes unacknowledged in this paper but would seem to be at the heart of the name disambiguation issue.*
- **Response: **Missing issue of ambiguous transliteration of names into roman alphabetic system has been added to the Introduction.
- *There are several approaches to name disambiguation but there are broadly two “schools”: attended and unattended. “Attended” is where humans interact with the disambiguation and “unattended” is where humans have no role. Attended disambiguation is the focus for identity management systems like ORCID - incentives need to be aligned for authors and others to participate in this and significant work goes into understanding the motivations and concerns of researchers in order to make these types of system successful. Unattended disambiguation makes use of the data that are available in the ecosystem (including the outputs of the attended disambiguation approach) to create a calculated output. We feel that the authors should give some of this background in their paper for context. In this context, it is important to acknowledge that only one identifier system has been successful in engaging the broad global academic community and that is ORCID. Other solutions are self-acknowledged proprietary solutions that aim to solve this problem in limited contexts (Researcherid.com/Publons to improve the Web of Science data (attended disambiguation); ScopusID - unattended disambiguation approach). Dimensions explicitly leverage ORCID data in an unattended person disambiguation approach. 1*
- **Response: **A short description of the unattended vs attended approaches of name disambiguation were added to the Introduction.
- *The authors claim that there is no standardization of unique identifiers for authors and documents.This is a very strong claim when viewed at an international ecosystem level. The majority of funders, publishers, institutions, scholarly societies and government agencies involved with research from around the world acknowledge DOIs as the key identifier for a research paper and ORCID as the principal identifier of researchers.*
- **Response: **We rewrote this statement to say that various other identifiers exist beyond the two most important ones, ORCID and DOI.
- *Some complexity lies in the authority that issues and maintains an article DOI (Crossref or DataCite in most cases but also, for example, J-Stage in Japan).*
* ORCID may currently be a “de facto standard” but the authors’ statement to this effect underplays the central role played by ORCID in the scholarly infrastructure community and the extent to which ORCID is the standard with which the majority of the commercial and open infrastructure providers engage. Google Scholar provides no API and makes no claim of persistence of identifiers; authors have limited control of their profile and of privacy. Researcherid.com (the progenitor of Publons/ResearcherID) is a founding member, supporter and participant of ORCID. We suggest that the authors should ensure that acknowledgement be made of ORCID’s suitability and level of adoption in academia. If the authors wish to note that ORCID is not the only standard, it would be appropriate to mention parallel efforts such as researchmap.jp in Japan and note that some countries remain reluctant to adopt ORCID as their principal identifier at this time.*
- **Response: **We have rephrased parts of the text to highlight the important role of ORCID.
- *The authors claim that it is not easy to determine the provenance of data in Dimensions has derived its data from ORCID or Publons. Arguably this is not the case. Dimensions clearly states that algorithmic methods are used with the input of ORCID data. 1 Publons/Researchid.com data is a proprietary source that could not be used in Dimensions without explicit acknowledgement of its use. *
- **Response: **We have removed this imprecisely-worded statement.
- *The authors further claim that no information is given about the completeness of these sources (e.g. Dimensions/Web of Science/Scopus), yet significant academic work has been undertaken to understand and benchmark coverage and completeness of these sources (see reference 2 for example). 2*
- **Response: **We have now removed claim about completeness of these sources since in our case, we are more interested in author-level completeness, e.g., are publications listed in Google scholar missing in ORCID for a specific author?
- *We agree with the authors that Open Science is indeed critical. This is why Dimensions, Web of Science, Scopus and others contain information on Open Access statuses of articles, provided by Unpaywall  ( https://unpaywall.org/integrations). Dimensions also contains full listings of preprint articles from many preprint servers including ArXiv.org, BioArXiv, the Center for Open Science, PeerJ and so on. As such we find the authors’ comment that these things are “not taken into account” to be misleading.*
- **Response: **We removed the imprecisely-worded claim about Dimensions’ open access status and preprints.
- *The authors then appear to contradict themselves by claiming that preprints and publications cause duplicate entries in these systems. In Dimensions, where the current reviewer has the most experience, publications from preprints are linked to final publications where this information is available, see for example https://app.dimensions.ai/details/publication/pub.1118864658?and_facet_researcher=ur.01123321343.51. We agree that manual intervention is often needed in these cases.*
- **Response: **This contradiction was removed by acknowledging the listing of preprints on other platforms, such as Dimensions. 
- *The authors claim that a free tool does not exist to explore metadata brought together from multiple sources, however, this does not acknowledge the rich lineage of free tools written to serve institutional use cases in author disambiguation and metadata aggregation including: VIVO ( https://duraspace.org/vivo/) and Catalyst Profiles ( http://profiles.catalyst.harvard.edu), both funded by the NIH; ImpactStory (e.g. https://profiles.impactstory.org/u/0000-0001-6728-7745/publications); and, ReCiter ( https://github.com/wcmc-its/ReCiter). The authors should endeavour to situate their work in the context of this prior work.*
- **Response: **We have added the above mentioned tools to the Introduction text and now describe our application in the context of those tools.
- *Symplectic Elements is, as the authors state, a commercial system focused on institutional use cases that meets this need. However, the authors fail to acknowledge that outside STEM subjects, coverage of research outputs becomes more challenging. The strength of commercial tools is often that, as they must meet institutional needs, work is put into diversifying coverage of different output types beyond the journals and conference proceedings that STEM subjects favour. While composing a faithful representation of a STEM academic and their output can be challenging from disparate data sources, doing the same for a non-STEM academic can be an order of magnitude more difficult. We believe that it is also fair to acknowledge that institutional engagement is not unimportant to improving the overall data quality in the bibliographic ecosystem.*
- **Response: **We have now added Introduction text, highlighting that commercial systems can make a big impact, especially in institutional cases.
- *The authors also appear to be unaware that Dimensions does bring records together from multiple sources including author information and that this is transparently documented in scholarly articles written by the Dimensions team, 1 as well as in system and API documentation ( https://docs.dimensions.ai/dsl/). Open access information and much more (citation information, funding information) and is available in the version of Dimensions that is available for free for personal use.*
- **Response: **We are aware of this functionality within Dimensions, but it is not open for use beyond personal use and most of the data that we are interested in is already in the public domain.
- *The authors note that Dimensions, Mendeley and others do not offer a public open API. While Dimensions does offer a free end-user tool, it does not offer a full open public API. However, Dimensions does offer a free metrics API for non-commercial purposes ( https://www.dimensions.ai/dimensions-apis/) and Mendeley continues to offer a free API (albeit with recent changes to some of the functionality around search functionality - https://dev.mendeley.com/).*
- **Response: **We now removed the imprecisely-worded statement about closed APIs of Dimensions and Mendeley.
- *In addition, given the use of Google Scholar by the authors in their tool, I think that it is important to note explicitly that Google Scholar does not offer an API. Indeed, from the software source code deposited by the authors, it appears that data is scraped from the Google Scholar website contravening the terms of use of Google Scholar. This fact should be explicitly noted in the paper.*
- **Response: **We have now added a statement that web scraping is done to retrieve information from Google Scholar and that this may contravene the terms of use of Google Scholar.  
- *The paper contains no critical analysis on the accuracy or success of the algorithmic approaches taken by the authors as regards fuzzy matching of papers and authors between sources.*
- **Response: **A description of the fuzzy matching mechanism that was applied was added together with a statement about accuracy of matching.
- *The paper would also be improved by including a flow diagram and description of matching approach taken by the authors.*
- **Response: **The description of the approach for matching of publications has been expanded. 
